# Chloroplast Phylogenomics and Barcode Discovery in Medicinal *Stachys* Species Reveal Evolutionary Relationships and Adaptive Signatures

**DOI:** 10.1002/ece3.73618

**Published:** 2026-05-03

**Authors:** Fatemeh Sadat Ghotbi, Aboozar Soorni

**Affiliations:** ^1^ Department of Biotechnology, College of Agriculture Isfahan University of Technology Isfahan Iran

**Keywords:** chloroplast microsatellites, comparative plastomics, phylogenetic, selective pressure, *Stachys*

## Abstract

The genus *Stachys*, a large and taxonomically intricate group within the Lamiaceae family, includes numerous species valued in traditional medicine, yet their evolutionary relationships are often obscured by morphological complexity. To resolve these persistent difficulties, we sequenced and assembled the complete chloroplast genomes of two medicinally important Iranian species, *Stachys persica* and *Stachys germanica* , and compared them against seven congeneric genomes. Despite an overall picture of strong structural conservation across the genus, we discovered that evolutionary variation concentrates in specific genomic hotspots. Several non‐coding intergenic spacers, most notably *trnL‐trnF*, along with the *matK* and *ycf1* genes emerged as exceptionally variable regions, making them promising candidates for species‐level DNA barcoding. In a more surprising finding, we detected clear signatures of positive selection acting on the photosynthetic gene *petB*, suggesting that even core energy pathways have experienced adaptive refinement within this lineage. Phylogenetically, whole‐plastome data placed *S. germanica* as sister to *S. byzantina* , with *S. persica* as their closest relative, a relationship that individual barcode loci failed to recover reliably. Taken together, this study provides a robust evolutionary framework for *Stachys*, identifies practical molecular tools for authenticating medicinal materials, and demonstrates the power of whole‐plastome sequencing to untangle taxonomically intricate plant groups.

## Graphical abstract

This study presents the first complete chloroplast genomes of two medicinally important *Stachys* species, *S. persica* and *S. germanica* . Comparative phylogenomic analyses revealed highly conserved genomes with lineage‐specific variations, identified hypervariable regions as candidate DNA barcodes, and detected signatures of positive selection in the petB gene. The robust phylogenomic framework clarifies evolutionary relationships and provides essential genomic resources for the taxonomy and conservation of this complex genus.

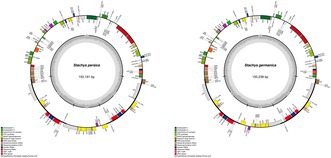

## 1 Introduction

The genus *Stachys* L., a large and cosmopolitan group comprising approximately 365 to 373 species globally, represents one of the most species‐rich and widely distributed genera within the Lamiaceae family (Salmaki et al. 2019; Tomou et al. 2020). The *Stachys* flora exhibits considerable morphological diversity and is sometimes problematic intraspecific morphological polymorphism, comprising annual and perennial herbs and subshrubs that occupy varied alpine, subalpine, and montane habitats (Lindqvist and Albert 2002). It has a subcosmopolitan but uneven distribution, with significant centers of diversity in the Mediterranean basin, Southwest Asia (notably Iran and Turkey), North and Central America, and Southern Africa (Güner et al. 2000; Kadereit 2004; Salmaki et al. 2019). This broad pattern is explained by a deep phylogenetic split into two major clades: one with an eastern Mediterranean origin that later expanded to Western Asia, Europe, and Africa, and another of Old World origin that subsequently diversified extensively in the Americas (Lindqvist and Albert 2002; Roy et al. 2013; Salmaki et al. 2012). Iran, situated within the critical Irano‐Turanian phytogeographical region, is recognized as a significant center of diversity for the genus, harboring 35 to 39 species (Rechinger 1982). Among its notable representatives are medicinally significant and ecologically widespread species such as *S. germanica* and *S. persica* (Salmaki et al. 2012), though *S. persica* is currently considered a synonym of *S. alpina* subsp. *alpina* according to Plants of the World Online (POWO).

Species of the genus *Stachys* hold significant value in traditional medicine, particularly within Middle Eastern folk phytotherapy, where various taxa are commonly prepared as infusions or decoctions to treat a spectrum of ailments, including infections, gastrointestinal disorders, inflammation, asthma, and skin diseases (Tomou et al. 2023, 2020). Phytochemical investigations have substantiated these uses, revealing that *Stachys* species produce a diverse array of bioactive secondary metabolites, including terpenoids, flavonoids, phenylethanoid glycosides, and phenolic acids, which are associated with broad pharmacological activities such as antioxidant, anti‐inflammatory, and antimicrobial effects (Goren et al. 2011; Tomou et al. 2023, 2020; Tundis et al. 2014). The essential oils of *Stachys* species further contribute to their bioactivity and are characterized by common volatile constituents such as β‐caryophyllene, germacrene D, and spathulenol, although composition varies significantly between species (Goren et al. 2011; Grujic‐Jovanovic et al. 2004; Lashgargahi and Shafaghat 2017). Focusing on the Iranian flora, *S. persica* has been studied for its essential oil, which is rich in non‐terpenoid compounds like methyl linoleate and hexadecanoic acid, as well as oxygenated sesquiterpenes (Khanavi et al. 2004). Research on *S. germanica* indicates its essential oil profile is often dominated by sesquiterpene hydrocarbons, particularly germacrene D (Razazi et al. 2023).

Accurate species identification is a cornerstone of biosystematics, yet the genus *Stachys* presents persistent taxonomic challenges. These difficulties stem from factors such as high morphological plasticity, overlapping cytological characteristics, and potential historical hybridization, which often obscure species boundaries and complicate classification based solely on traditional markers (Mulligan and Munro 1989; Salmaki et al. 2012, 2008). While morphological traits (e.g., calyx and corolla structure) and palynological studies offer some diagnostic value, they have proven insufficient for resolving complex interspecific relationships within this polymorphic genus (Berumen Cornejo et al. 2017). For instance, palynological studyacross numerous Iranian *Stachys* taxa could not provide definitive diagnostic separation for all species, highlighting the limitations of these approaches (Salmaki et al. 2008). Consequently, molecular tools have become indispensable for elucidating *Stachys* phylogeny. Early fingerprinting techniques such as ISSR and RAPD lacked the genomic depth and phylogenetic resolution required to robustly distinguish closely related species or clarify recent evolutionary events (Kharazian et al. 2015; Kochieva et al. 2006). Subsequently, phylogenetic frameworks based on more informative markers, including the nuclear ITS region and shorter cp DNA fragments (e.g., *trnL‐F*, *rps16*), successfully established the monophyly of the Stachydeae tribe and revealed its deep divergence into two major biogeographic lineages (Berumen Cornejo et al. 2017; Dündar et al. 2013; Roy et al. 2013; Salmaki et al. 2012; Seyedipour et al. 2017). However, these standard loci often provide insufficient variation to fully resolve species‐level relationships. To address these persistent limitations and advance the systematics of critical species, the high‐resolution approach of complete cp genome sequencing is necessary.

Chloroplast (cp) genomes in flowering plants are compact, structurally conserved, and highly stable, making them valuable for genetic variation analysis, evolutionary studies, and species identification via DNA markers (Daniell et al. 2016; Dobrogojski et al. 2020; Ravi et al. 2008). These attributes have advanced research on plant domestication, historical biogeography, and taxonomic clarification across genera and families (Jaswal et al. 2025; Zhang et al. 2023). In contemporary botanical research, cp genome data are increasingly used to clarify taxonomic relationships across plant genera and broader family groupings (Dong et al. 2015, 2012). This utility is powerfully demonstrated within the large and taxonomically complex mint family, Lamiaceae. For example, a landmark phylogenomic study by Zhao et al. (2021) leveraged 79 plastid protein‐coding genes from 175 species to construct a robust tribal‐level backbone for the family. This work established a modern classification framework of 12 subfamilies and 22 tribes, setting a new standard for understanding mint evolution and providing a critical reference for subsequent genus‐level studies (Zhao et al. 2021). In line with this phylogenetic renaissance, cp genomes are now routinely employed to elucidate relationships within individual Lamiaceae genera. For instance, studies on *Satureja* (Diani Gohar and Soorni 2025), *Mentha* (Soorni and Golchini 2025), *Salvia* (Akrami et al. 2025), and *Teucrium* (Hejazi et al. 2025) have successfully utilized whole cp genome data to clarify infrageneric phylogenies, confirm monophyly, and propose candidate DNA barcodes such as *ycf1* and *ndhF*. Notwithstanding this progress, the genus *Stachys* (tribe Stachydeae) remains comparatively understudied at the genomic level. While foundational phylogenetic placement has been confirmed, existing publications on *Stachys* cp genomes are largely limited to brief reports presenting basic assembly and annotation data for individual species, such as *S. japonica* (Wang et al. 2020), *S. sieboldii* (Huang et al. 2020), and *S. geobombycis* (Wang, Lan, et al. 2024). Although the study by Wang, Lan, et al. (2024) included a comparative analysis of eight species and identified several variable regions, a comprehensive and detailed comparative genomic investigation across a broader phylogenetic spectrum of *Stachys* is still lacking. A critical manifestation of this gap is the complete lack of cp genome sequences for specific and phylogenetically informative species, including *S. persica* and *S. germanica* . Without these genomic blueprints, it is impossible to determine their precise evolutionary placement within *Stachys* or to evaluate genome‐wide patterns of mutation, selection, and structural evolution that distinguish them from congeners. Addressing this deficit by sequencing these species will directly enable the identification of unique molecular signatures, clarify taxonomic boundaries, and provide the data required for high‐resolution phylogenetic studies that can inform conservation priorities for these and related species.

Hence, we sequenced the complete cp genomes of *S. persica* and *S. germanica* to address this critical data gap. This study aimed to: (i) assemble, annotate, and characterize the first complete cp genomes of these two species; (ii) perform a comparative genomic analysis to examine codon usage bias, structural dynamics at inverted repeat boundaries, and the distribution of simple sequence repeats (SSRs); (iii) assess nucleotide diversity and identify protein‐coding genes under positive selection; and (iv) reconstruct a robust phylogenomic framework to determine the precise evolutionary placement of *S. persica* and *S. germanica* within the genus *Stachys*. Furthermore, we evaluated the phylogenetic utility of individual candidate barcode loci (*matK*, *ycf1*, and *rpl16*) against whole‐genome data.

## 2 Materials and Methods

### 2.1 Plant Material and DNA Sequencing

Fresh, healthy leaf material was obtained from two distinct *Stachys* species, *S. persica* and *S. germanica* , collected during their active flowering phase to ensure optimal DNA yield. Total genomic DNA was extracted from 100 mg of cryopreserved leaf tissue using the DNeasy Plant Mini Kit (QIAGEN, Hilden, Germany), adhering to the manufacturer's protocol. The integrity of the DNA was verified by electrophoresis on a 1% agarose gel, confirming the presence of high‐molecular‐weight fragments. Purity and concentration were precisely measured with a NanoDrop 2000c spectrophotometer (Thermo Fisher Scientific, USA), with samples retained for further processing only if they met stringent purity criteria (A260/A280 ≥ 1.8; A260/A230 ≥ 2.0). Sequencing libraries were prepared from these high‐quality DNA extracts using the Illumina TruSeq DNA kit, targeting an average insert size of 350 bp. Paired‐end sequencing (2 × 150 bp) was subsequently carried out on an Illumina HiSeq 2000 platform (Illumina Inc., USA).

### 2.2 Cp Genome Reconstruction and Annotation

The raw paired‐end reads generated for each species first underwent a stringent quality assessment with FastQC (v0.11.9). Preprocessing was performed with Trimmomatic (Bolger et al. 2014) to eliminate adapter contaminants and trim low‐quality nucleotide regions, yielding a refined set of high‐fidelity reads. De novo assembly of the complete cp genomes was conducted using GetOrganelle v1.7.7.1 (Jin et al. 2020), which leverages a baiting and iterative mapping strategy specific to organelle genomes. The topological correctness and completeness of the resulting assembly graphs were meticulously validated through visual inspection in Bandage (Wick et al. 2015). Assembly accuracy was further validated by mapping the trimmed reads back to the assembled cp genomes using Bowtie2 (Langmead et al. 2009) with default parameters, and coverage depth was assessed using Samtools (Li et al. 2009). For comprehensive annotation, the assembled genomes were analyzed in parallel with CPGAVAS2 (Shi et al. 2019) and GeSeq (Tillich et al. 2017), enabling precise demarcation of genomic features such as genes, transfer RNAs, and ribosomal RNAs. Finally, professional‐grade circular diagrams of the annotated cp genomes were constructed for publication using OGDRAW (Greiner et al. 2019).

### 2.3 Analysis of Codon Usage Bias

Synonymous codon usage patterns were examined to assess biases in cp genomes of *S. persica* and *S. germanica* . Relative Synonymous Codon Usage (RSCU) values were computed for all shared protein‐coding genes. This calculation was performed using the codon usage analysis module implemented in MEGA6 (Tamura et al. 2013). The resulting RSCU profiles for both species were subsequently visualized and compared graphically to identify taxa‐specific codon preference patterns using the RSCU‐Plot Shiny application (https://pcg‐lab.shinyapps.io/RSCU‐Plot/; Akrami et al. 2025; Diani Gohar and Soorni 2025; Hejazi et al. 2025; Soorni and Golchini 2025).

### 2.4 Comparative Cp Genome Analysis and IR Boundary Assessment

A structural comparison of cp genomes was conducted to investigate large‐scale evolutionary dynamics. The focus was on the characterization of the Inverted Repeat (IR) boundaries and their adjacency to the Large and Small Single‐Copy (LSC, SSC) regions. The precise junctions (JLB, JSB, JSA, JLA) between these regions were mapped for both *S. persica* and *S. germanica* and seven additional species retrieved from the NCBI database (NC_062803 ( *S. affinis* ), NC_029825 ( *S. byzantina* ), NC_029822 ( *S. chamissonis* ), NC_029823 ( *S. coccinea* ), NC_082543 (*S. geobombycis*), MT554703 ( *S. japonica* ), NC_029824 ( *S. sylvatica* )). At the time of analysis, these seven represented all complete and well‐annotated *Stachys* chloroplast genomes publicly available. Several additional accessions were examined but were excluded due to incomplete annotation or questionable assembly quality, which would have compromised the comparative analyses. The visualization of these border regions and the associated genes was achieved using the web‐based tool IRScope (Amiryousefi et al. 2018).

### 2.5 Identification of Simple Sequence Repeats (SSRs)

The prevalence and distribution of SSRs, or microsatellites, were characterized in cp genomes of nine species. The identification was performed using the CPStools (Huang et al. 2024) tool with stringent parameters to ensure robust detection. The search criteria were defined as follows: a minimum of 10 repeat units for mononucleotides, 6 for dinucleotides, 5 for trinucleotides, and 4 repeat units for tetra‐, penta‐, and hexanucleotide motifs. The results were categorized by repeat type and genomic location (gene/IGS) to provide a comprehensive overview of the mutational hotspot landscape and to identify potential polymorphic markers for future population‐level studies in the genus *Stachys*.

### 2.6 Assessment of Nucleotide Diversity and Selective Pressure

Genetic divergence and evolutionary constraints acting on the protein‐coding genes of *S. persica* and *S. germanica* were evaluated. Nucleotide diversity (Pi) was calculated across the aligned cp genomes using a sliding window approach (window length: 800 bp; step size: 200 bp) in DnaSP v6 (Rozas et al. 2017) to quantify sequence polymorphism and identify hypervariable regions. Because Pi values varied considerably across species and genera, hypervariable regions were defined empirically as the five regions with the highest Pi values for each CDS and IGS region. Concurrently, to detect signatures of diversifying selection, we implemented a suite of codon‐based site models. Statistical comparisons of nested model pairs (M0/M3, M1a/M2a, M7/M8, M8a/M8) were performed within the EasyCodeML (Gao et al. 2019) framework, with likelihood ratio tests (LRTs) applied to determine significance (*p* < 0.05). This approach allowed for the identification of protein‐coding genes evolving under non‐neutral evolution. For these genes, we inferred specific amino acid residues under positive selection based on a Bayes Empirical Bayes (BEB) analysis, which identifies sites with a high posterior probability (*p* > 0.95) of having a ω (dN/dS) ratio > 1.

### 2.7 Phylogenetic Reconstruction

A phylogenetic framework for *S. persica* and *S. germanica* was established based on a comprehensive cp genome dataset. The taxon sampling encompassed the target species, representatives from some major Lamiaceae genera, and *Ajuga bracteosa* as the outgroup (Diani Gohar and Soorni 2025; Hejazi et al. 2025; Soorni and Golchini 2025). Data processing involved: (i) individual alignment of coding sequences with MUSCLE v3.8.1551 (Edgar 2004); (ii) refinement of alignments using trimAl v1.4 (Capella‐Gutiérrez et al. 2009) with stringent parameters to exclude ambiguous sites; and (iii) concatenation of the filtered alignments into a supermatrix using SequenceMatrix (Vaidya et al. 2011). Model selection via ModelFinder identified GTR + F + I + G4 as the most appropriate substitution model. The maximum likelihood tree was computed in IQ‐TREE (Nguyen et al. 2015), with statistical support for nodes derived from 1000 ultrafast bootstrap replicates. The final topology was visualized and graphically refined in iTOL (Letunic and Bork 2021).

### 2.8 Evaluation of Marker Resolution

To assess the suitability of specific loci for phylogenetic barcoding, the protein‐coding genes *matk*, *ycf1*, *rpl16*, and combination of them were analyzed separately. These markers were subjected to the same analytical workflow, alignment, trimming, and phylogenetic inference to determine their relative power to resolve relationships within and among *Stachys* species.

## 3 Results

### 3.1 Features of *Stachys* Cp Genomes

**FIGURE 1:**
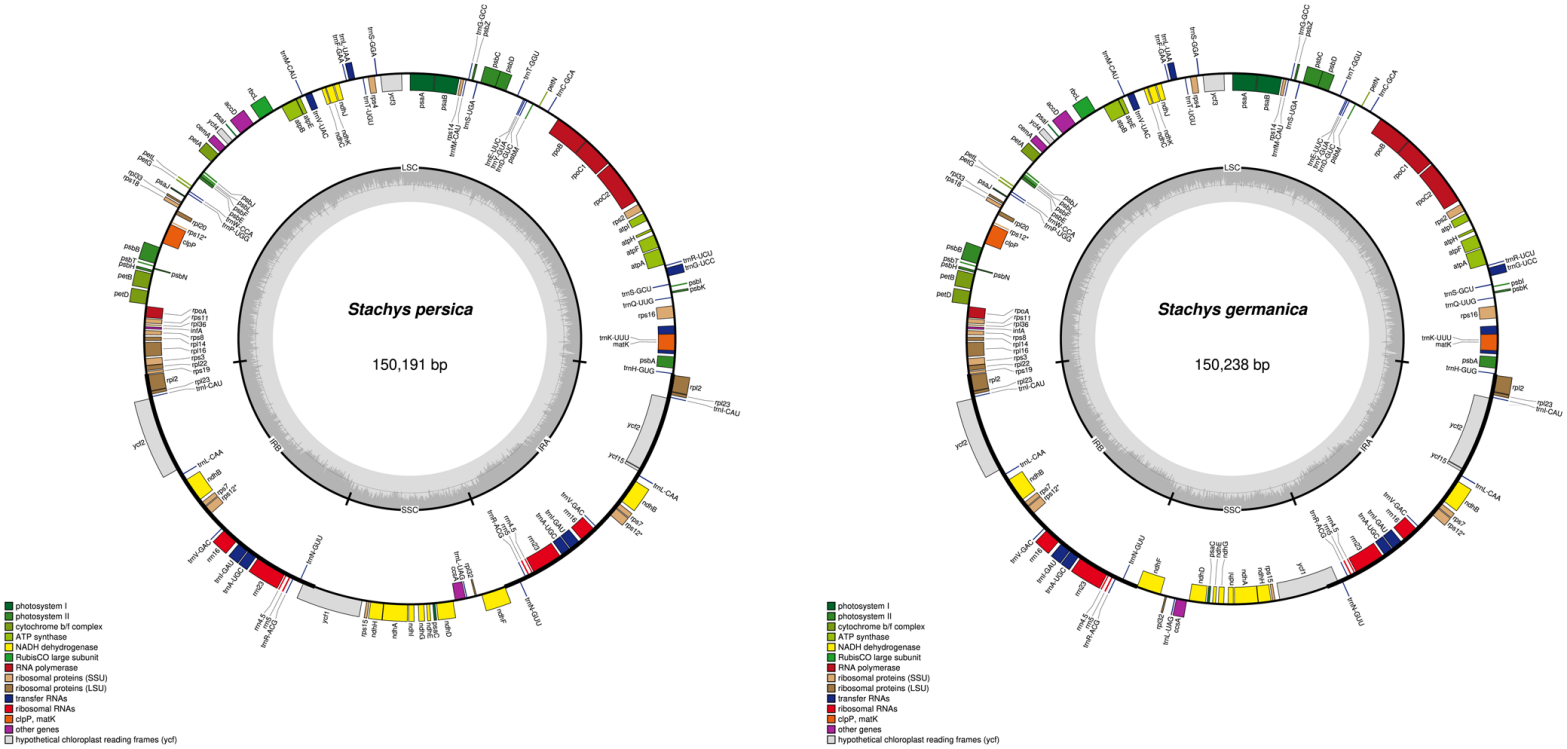
Circular gene map of the chloroplast genomes of two *Stachys* species. The diagram, generated using OGDRAW, illustrates the conserved quadripartite structure. From the center outward: the first circle shows the two inverted repeat regions (IRa and IRb) in light gray, which separate the large single‐copy (LSC) and small single‐copy (SSC) regions. The second circle represents the GC content, where darker peaks indicate higher GC content. The third circle visualizes the GC skew, using green and purple colors to denote positive and negative skew, respectively. The outermost circle displays the gene features, with genes color‐coded by their functional groups (e.g., photosynthesis, self‐replication). Genes shown on the inside of this circle are transcribed clockwise, while those on the outside are transcribed counter clockwise.

A total of 2,251,122 high‐quality paired‐end reads were generated for *S. persica* and 2,187,214 for *S. germanica* after trimming. Assembly of these reads produced complete circular cp genomes with average coverage depths of 168× for *S. persica* and 152× for *S. germanica* . Both genomes showed the typical quadripartite structure found in most flowering plants (Figure 1). When the junction points between the single‐copy and inverted repeat regions were compared, a small but consistent difference in length emerged. The LSC region of *S. germanica* was 42 bp longer than that of *S. persica* (81,491 bp versus 81,449 bp). This difference carried through to the rest of the genome. The IRb region measured 107,126 bp in *S. germanica* and 107,086 bp in *S. persica* , while IRa measured 150,238 bp and 150,191 bp, respectively. The SSC region was also slightly longer in *S. germanica* (17,477 bp) than in *S. persica* (17,468 bp). As a result, the complete plastome of *S. germanica* (150,238 bp) was 47 bp longer than that of *S. persica* (150,191 bp). Visual inspection of the assemblies using Bandage showed clean, circular graphs for both genomes, with no extra branches or ambiguous nodes. To double‐check the assemblies, the cleaned reads were mapped back to each genome using Bowtie2. More than 92% of the reads mapped successfully, and coverage remained steady across the LSC, SSC, and both IR regions.

A comprehensive annotation of genes further elucidated the functional composition of the two plastomes. The investigation categorized the 131 identified genes into distinct functional groups for both species (Table 1). Genes dedicated to photosynthetic machinery constituted a significant portion, with 15 genes for photosystem II subunits, five for photosystem I subunits, six for the cytochrome b6/f complex, 12 for NADH dehydrogenase subunits, six for ATP synthase subunits, and the single large subunit of Rubisco, yielding a total of 45 genes. The self‐replication apparatus was similarly conserved, comprising 11 genes for large ribosomal proteins, 14 for small ribosomal proteins, four for RNA polymerase subunits, eight ribosomal RNAs (duplicated in the IR regions), and 37 transfer RNAs, culminating in 74 genes. The remaining 12 genes, categorized under other functions, included *matK*, *clpP*, *cemA*, *accD*, *ccsA*, *infA*, and several conserved open reading frames (*ycf* genes), with no discrepancy in presence or copy number observed between the two species. Critically, the analysis confirmed that the minor difference in overall genome size was not attributable to the gain, loss, or duplication of any functional gene, but rather to expansion or contraction in the non‐coding intergenic regions.

**TABLE 1:** Gene content and functional classification of two *Stachys* cp genomes. Genes are categorized by functional groups, with intron‐containing genes (#) and multi‐copy genes (n) indicated.

Category	Gene group	Gene name	*S. persica*	*S. germanica*
Photosynthesis	Subunits of photosystem I	*psaB, psaA, psaI, psaJ, psaC*	5	5
	Subunits of photosystem II	*psbA, psbK, psbI, psbM, psbD, psbC, psbZ, psbJ, psbL, psbF, psbE, psbB, psbT, psbN, psbH*	15	15
	Subunits of NADH dehydrogenase	*ndhJ, ndhK, ndhC, ndhB(2)#, ndhH, ndhA#, ndhI, ndhG, ndhE, ndhD, ndhF*	12	12
	Subunits of cytochrome b/f complex	*petN, petA, petL, petG, petB#, petD#*	6	6
	Large subunit of rubisco	*rbcL*	1	1
	Subunits of ATP synthase	*atpA, atpF#, atpH, atpI, atpE, atpB*	6	6
Self‐replication	Proteins of large ribosomal subunit	*rpl33, rpl20, rpl36, rpl14, rpl16#, rpl22, rpl2(2)#, rpl23(2), rpl32*	11	11
	Proteins of small ribosomal subunit	*rps12(2)##, rps16#, rps2, rps14, rps4, rps18, rps11, rps8, rps3, rps19, rps7(2), rps15*	14	14
	Subunits of RNA polymerase	*rpoC2, rpoC1#, rpoB, rpoA*	4	4
	Ribosomal RNAs	*rrn16(2), rrn23(2), rrn4.5(2), rrn5(2)*	8	8
	Transfer RNAs	*trnH‐GUG, trnK‐UUU#, trnQ‐UUG, trnS‐GCU, trnG‐UCC#, trnR‐UCU, trnC‐GCA, trnD‐GUC, trnY‐GUA, trnE‐UUC, trnT‐GGU, trnS‐UGA, trnG‐GCC, trnfM‐CAU, trnS‐GGA, trnT‐UGU, trnL‐UAA#, trnF‐GAA, trnV‐UAC#, trnM‐CAU, trnW‐CCA, trnP‐UGG, trnI‐CAU(2), trnL‐CAA(2), trnV‐GAC(2), trnI‐GAU(2)#, trnA‐UGC(2)#, trnR‐ACG(2), trnN‐GUU(2), trnL‐UAG*	37	37
Other genes	Maturase	*matK*	1	1
	Protease	*clpP##*	1	1
	Envelope membrane protein	*cemA*	1	1
	Acetyl‐CoA carboxylase	*accD*	1	1
	c‐type cytochrome synthesis gene	*ccsA*	1	1
	Translation initiation factor	*infA*	1	1
	Conserved open reading frames	*ycf3##, ycf4, ycf2(2), ycf1, ycf15*	6	6
Total			131	131

### 3.2 Comparative Analysis of Codon Usage Bias

**FIGURE 2:**
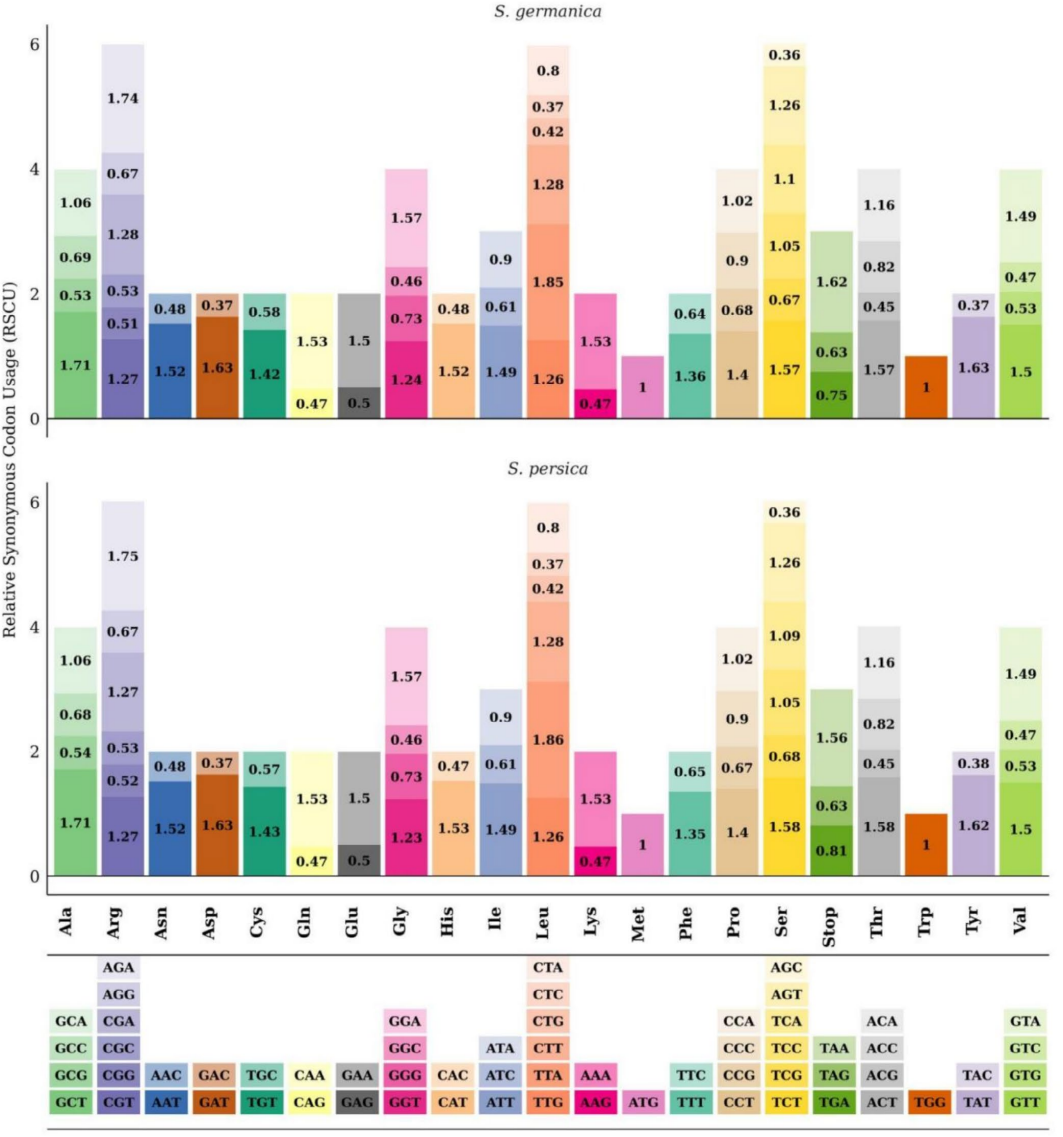
The bar plot of the Relative Synonymous Codon Usage (RSCU) values for each amino acid, grouped by species ( *S. persica* and *S. germanica* ). Codons are color‐coded, and their corresponding amino acids are labeled below the plot.

The evaluation of synonymous codon preference in cp genomes of *S. germanica* and *S. persica* demonstrated a profound concordance in their usage patterns, underpinned by a clear preferential use of nucleotides A and T in the third codon position. The RSCU profiles of the two species were virtually superimposable, with the values for 57 of the 64 codons being perfectly identical, leaving only a minimal set of seven codons exhibiting a marginal divergence of merely 0.01 RSCU units (Figure 2). Notably, the most substantial variation was observed in the stop codon family, where the RSCU for TGA was 0.75 in *S. germanica* compared to 0.81 in *S. persica* , and for TAA, it was 1.62 versus 1.56, respectively. Other minor fluctuations included codons TGT (Cys), CAT (His), TTA (Leu), TCT (Ser), TCA (Ser), and ACT (Thr), none of which altered the overall high‐usage status of A/T‐ending synonyms or the low‐usage of G/C‐ending ones. This exceptional degree of conservation indicates that the evolutionary forces governing codon selection in cp genomes of these two *Stachys* species are exceptionally stable and consistent.

### 3.3 Structural Variation at IR Boundaries

**FIGURE 3:**
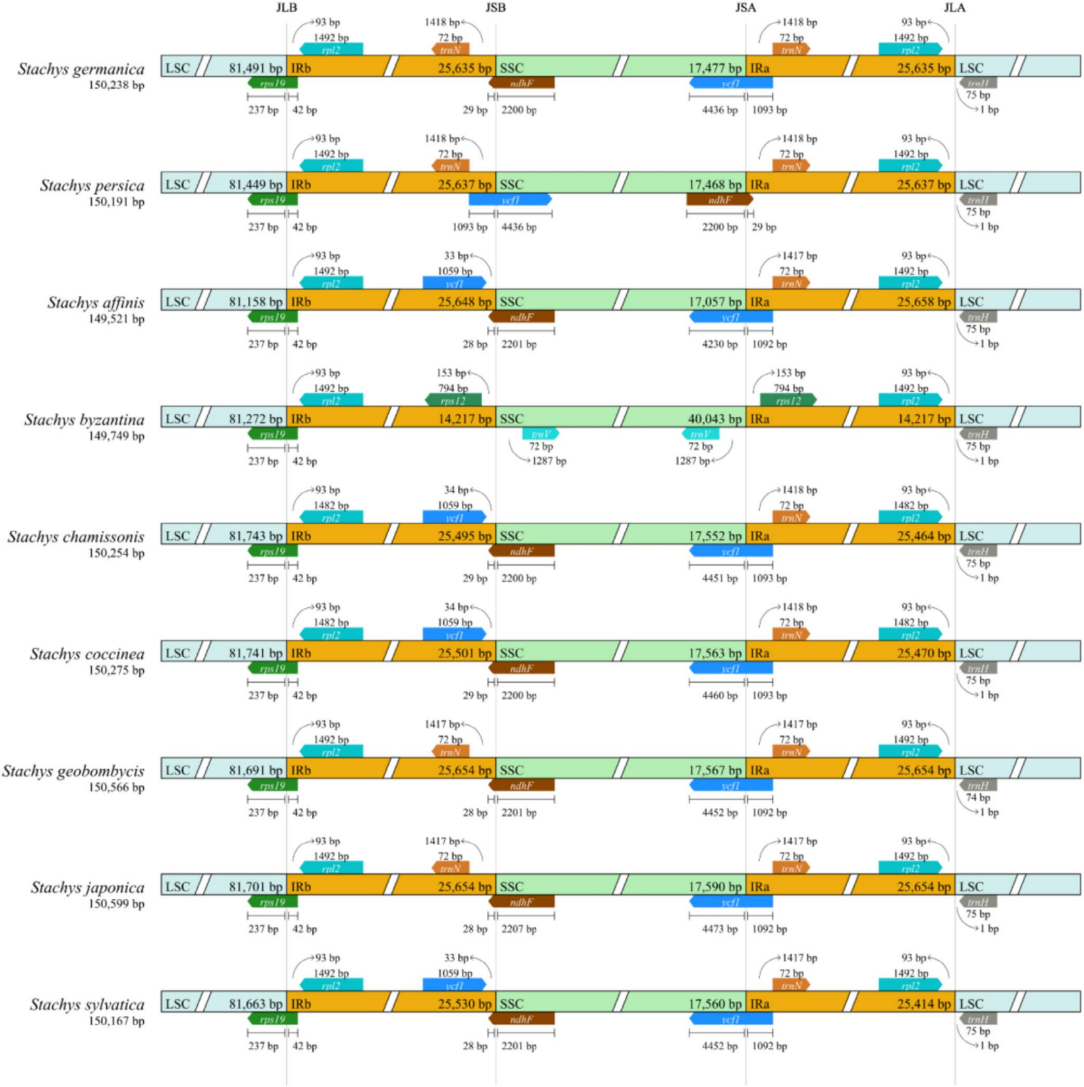
Comparison of the boundaries of the large single‐copy (LSC), small single‐copy (SSC), and inverted repeat (IR) regions among three *Stachys* species. Key junctions are labeled: JLB (LSC/IRb), JSB (SSC/IRb), JSA (SSC/IRa), and JLA (LSC/IRa), highlighting structural variations at the IR‐LSC and IR‐SSC borders.

Based on a comprehensive analysis of cp genomes of nine *Stachys* species, including the newly sequenced *S. persica* and *S. germanica* , this study revealed significant structural divergence, particularly in the size of the inverted repeat (IR) regions and the positioning of genes at their boundaries, underscoring a dynamic evolutionary history within the genus (Figure 3). The overall cp genome size ranged from 149,521 bp in *S. affinis* to 150,599 bp in *S. japonica* . A striking anomaly was observed in *S. byzantina* , which exhibited a dramatic contraction of its IR regions (14,217 bp each) compared to the other species (ranging from ~25,414 to ~25,658 bp). This contraction was accompanied by a compensatory expansion of its SSC region to 40,043 bp, whereas the SSC in other species varied between 17,057 bp ( *S. affinis* ) and 17,590 bp ( *S. japonica* ). The LSC region was more conserved in length, ranging from 81,158 bp ( *S. affinis* ) to 81,743 bp ( *S. chamissonis* ).

The distribution and length of genes spanning the junction sites further highlighted interspecific variations. The *rps19* gene was universally conserved at the JLB junction, with a 237 bp segment in the LSC and a 42 bp extension into the IRb. In contrast, the positioning of other key genes differed. The *rpl2* gene was located entirely within the IRb and IRa in all species, with a length of 1492 bp in most, except for *S. chamissonis* and *S. coccinea* , where it was slightly shorter (1482 bp). The *trnN* gene (72 bp) was also consistently found within the IRs. A major point of divergence involved the *ndhF* and *ycf1* genes. In species like *S. germanica* , *S. geobombycis*, and *S. japonica* , *ndhF* spanned the JSB boundary with a small segment (28–29 bp) in the IRb and a large segment (2200–2207 bp) in the SSC. Conversely, in *S. persica* , this arrangement was inverted. Similarly, the *ycf1* gene exhibited considerable length polymorphism and positional shifts. Its total length varied from 5322 bp in *S. affinis* to 5565 bp in *S. japonica* . In most species, a truncated *ycf1* pseudogene (~1059 bp) was located entirely within the IRb, while the functional copy spanned the JSA boundary. However, *S. byzantina* lacked these typical IR/SSC junction genes entirely, instead featuring a unique structure with *rps12* in the IRs and duplicated *trnF* genes in the SSC. These detailed comparisons of gene content and boundary shifts provide crucial insights into the molecular evolution and phylogenetic relationships within the *Stachys* genus.

### 3.4 cpMicrosatellite Distribution in *Stachys* Species

Comprehensive analysis of cp genomes across nine *Stachys* species, with particular focus on *S. persica* and *S. germanica* , revealed distinct patterns of cpSSR distribution that highlight both conserved genomic features and species‐specific differentiation. The survey identified 27 and 23 cpSSRs in *S. persica* and *S. germanica* , respectively, positioning them near the mean abundance observed across the genus (range: 23–30 SSRs; mean: 26.1).

Mononucleotide A/T repeats predominated the SSR composition in both focal species, representing 19 (70.4%) of the repeats in *S. persica* and 16 (69.6%) in *S. germanica* . This distribution aligns with the characteristic AT‐rich nature of cp genomes. Notably, dinucleotide repeats were equally represented in both species with 2 occurrences each (7.4% in *S. persica* , 8.7% in *S. germanica* ). A significant distinction emerged in complex repeat motifs: *S. persica* contained 6 trinucleotide repeats (22.2%), while *S. germanica* possessed 5 (21.7%), reflecting subtle differences in SSR architecture.

**FIGURE 4:**
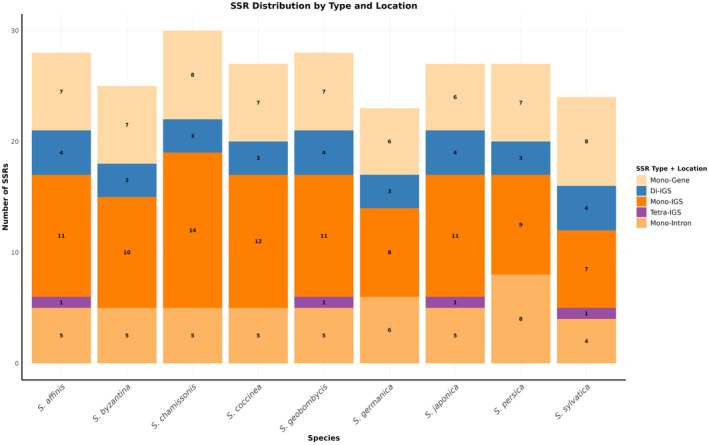
The types and distribution of SSRs along the chloroplast genomes of *Stachys* species.

The genomic localization of SSRs exhibited a strong bias toward non‐coding regions across all examined species (Figure 4). In *S. persica* , 17 SSRs (63.0%) resided in intergenic spacers (IGS), 4 (14.8%) in intronic regions, and 6 (22.2%) within gene coding sequences. Similarly, *S. germanica* displayed 14 IGS‐located SSRs (60.9%), 4 intronic (17.4%), and 5 genic (21.7%). This consistent pattern confirms IGS regions as primary sites for microsatellite accumulation in *Stachys* cp genomes.

Comparative analysis of specific IGS regions revealed loci with considerable phylogenetic significance. The trnS‐GCU‐trnG‐UCC_1 IGS emerged as a conserved hotspot, containing identical 7‐base TA repeats in both *S. persica* and *S. germanica* —a motif also conserved in *S. japonica* and *S. geobombycis*. However, the trnE‐UUC‐trnT‐GGU IGS demonstrated species‐specific variation, with *S. persica* featuring an unusual 9‐base TA repeat compared to the 6‐base repeat in *S. germanica* .

The atpF_1‐atpH IGS further highlighted interspecific differences. This region contained two adjacent SSRs (T12 and A11) in *S. persica* , whereas *S. germanica* featured only a single T10 repeat at this locus. The ycf1 gene, a known variable region, also displayed distinct patterns: *S. persica* contained two A10 repeats within this gene, while *S. germanica* featured two T10 repeats at different positions, suggesting independent evolutionary trajectories.

Several loci demonstrated remarkable conservation across the genus. The *matK* gene uniformly contained a T12 mononucleotide SSR in all nine species. Similarly, the *atpB* gene consistently featured a T10 repeat, and the infA‐rps8 IGS maintained SSR presence across all taxa, indicating strong evolutionary constraint at these positions.

In summary, cpSSR analysis provides clear molecular discrimination between *Stachys* species. The profiles of *S. persica* and *S. germanica* share fundamental characteristics of SSR distribution but are distinguished by specific variations in repeat number, complex motif composition, and precise genomic localization. These species‐specific signatures, particularly in the trnE‐UUC‐trnT‐GGU IGS and *ycf1* gene regions, offer valuable molecular markers for phylogenetic reconstruction and species delineation within the genus.

### 3.5 Widespread and Intense Signatures of Positive Selection in Plastid Genes

The analysis of positive selection within *Stachys* species revealed a more restricted signature of positive selection. Among all plastid genes examined, only *petB*, which encodes the cytochrome *b*_*6*_ subunit of the cytochrome *b*_*6*_ *f* complex, exhibited statistically significant evidence of site‐specific positive selection. The M8 model, which allows for a class of sites with ω > 1, was strongly favored over the neutral M7 model (LRT *p* = 5.24 × 10^−6^). This model identified two codons under positive selection with high posterior probabilities: a leucine (L) at the very first site of the mature protein (site 1, posterior probability = 0.997**) and an asparagine (N) at site 2 (posterior probability = 0.961). The strength of the signal at the N‐terminal region suggests that adaptive evolution in *Stachys* may be focused on critical structural or interaction interfaces of the cytochrome *b*_*6*_ *f* complex, a key component of the photosynthetic electron transport chain. The absence of significant positive selection signals in other core plastid genes in *Stachys* indicates a lineage‐specific adaptive process centered on the optimization of photosynthetic electron flow.

### 3.6 Nucleotide Diversity Across Cp Genome of *Stachy*s Species

**FIGURE 5:**
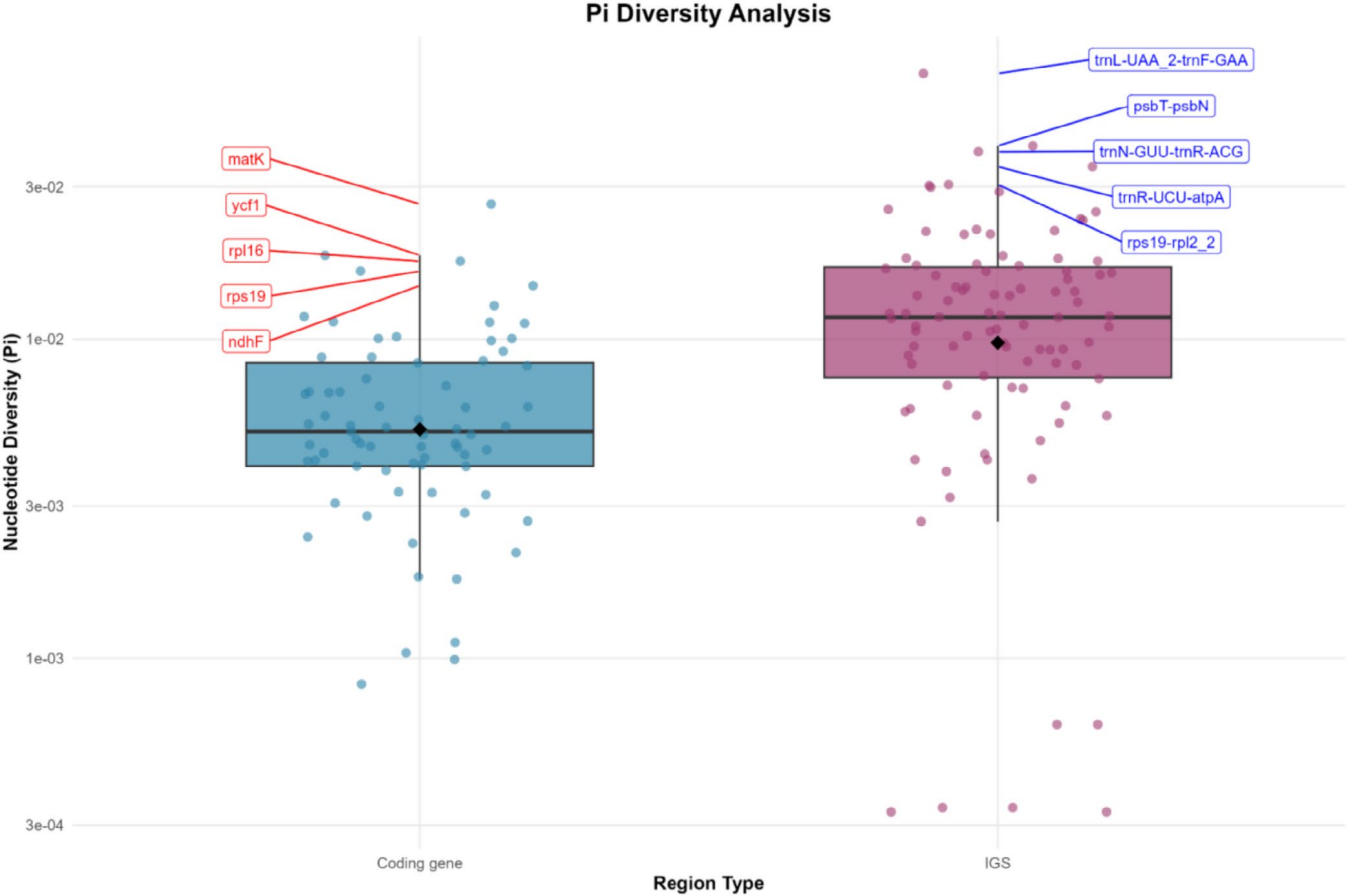
Comparative analysis of nucleotide diversity (Pi) in *Stachys* cp genomes. Boxplots depict Pi values for coding genes (blue) and intergenic spacer regions (IGS; orange), with jittered points showing individual values. The top and bottom three most extreme values for each region type are labeled with feature names and exact Pi values. White diamonds indicate mean diversity values.

Analysis of nucleotide diversity (Pi) across cp genomes of nine *Stachys* species, including the newly sequenced *S. germanica* and *S. persica* , revealed a spectrum of evolutionary constraints, ranging from absolute conservation to high variability (Figure 5). A significant number of regions were found to be entirely invariant (Pi = 0), demonstrating perfect sequence conservation across all 10 species. These included the coding genes *psbE*, *psbI*, *psbL*, *psbN*, *psbT*, *ycf15*, and the tRNA genes *trnC‐GCA, trnD‐GUC, trnE‐UUC, trnF‐GAA, trnG‐GCC, trnH‐GUG, trnK‐UUU, trnL‐CAA, trnL‐UAA, trnL‐UAG, trnM‐CAU, trnN‐GUU, trnQ‐UUG, trnR‐ACG, trnR‐UCU, trnS‐GCU, trnS‐GGA, trnS‐UGA, trnT‐GGU, trnT‐UGU, trnV‐GAC, trnW‐CCA, and trnY‐GUA*, as well as the protein‐coding gene *petN*. Furthermore, several intergenic spacers also showed no variation: *ndhB_1‐rps7, ndhB_2‐trnL‐CAA, ndhK‐ndhC, psaB‐psaA, psbF‐psbE, rpl23‐rpl2_1, rpl2_1‐rpl23, rpoC1_1‐rpoB, rps12_2‐rps12_3, rps12_3‐rps12_2, rps7‐ndhB_1, and ycf15‐ycf2*.

Beyond these invariant sequences, the variable regions exhibited Pi values spanning nearly two orders of magnitude from 0.00033 to 0.06828. Among the 71 variable protein‐coding genes analyzed, the maturase‐encoding gene *matK* was the most polymorphic (Pi = 0.02649), consistent with its established role as a plant barcode. Other notably diverse coding genes included *ycf1* (Pi = 0.01831), *rpl16* (Pi = 0.01755), *ndhF* (Pi = 0.01471), and several genes involved in cytochrome c synthesis (*ccsA*, Pi = 0.01177) and ribosomal large subunits (*rpl33*, Pi = 0.01133; *rpl36*, Pi = 0.01121). In stark contrast, several genes exhibited extreme conservation, indicative of strong functional constraints. These included the ribosomal protein gene *rps7* (Pi = 0.00083), the NADH dehydrogenase gene *ndhB* (Pi = 0.00112), and the genes *petD* (Pi = 0.00104) and *ycf3* (Pi = 0.00099).

As anticipated, variable intergenic spacer regions generally displayed higher nucleotide diversity than coding sequences, underscoring the lower selective constraint on non‐functional DNA. The most variable region in the entire plastome was the IGS trnL‐UAA_2‐trnF‐GAA (Pi = 0.06828). Several other IGS regions constituted pronounced peaks of diversity, including psbT‐psbN (Pi = 0.04033), trnN‐GUU‐trnR‐ACG (Pi = 0.03864), trnR‐UCU‐atpA (Pi = 0.03472), and the common barcoding spacer trnH‐GUG‐psbA (Pi = 0.02991). Conversely, some IGS regions were remarkably conserved, particularly those likely residing within the inverted repeat regions, such as ndhB_1‐ndhB_2 and rpl2_1‐rpl2_2 (both Pi = 0.00033), where efficient copy‐correction through gene conversion suppresses sequence divergence.

A comparative overview confirms that the hypervariable hotspots of the *Stachys* cp genome are overwhelmingly concentrated in IGS regions. The top 10 most variable loci were exclusively IGS, with the exception of the *matK* and *ycf1* genes. This pattern, starkly contrasted by the extensive set of invariant sequences, highlights the particular utility of specific non‐coding spacers such as trnL‐UAA_2‐trnF‐GAA, psbT‐psbN, and trnH‐psbA for developing high‐resolution molecular markers for phylogenetic and population genetic studies within the genus.

### 3.7 Phylogenetic Analysis

**FIGURE 6:**
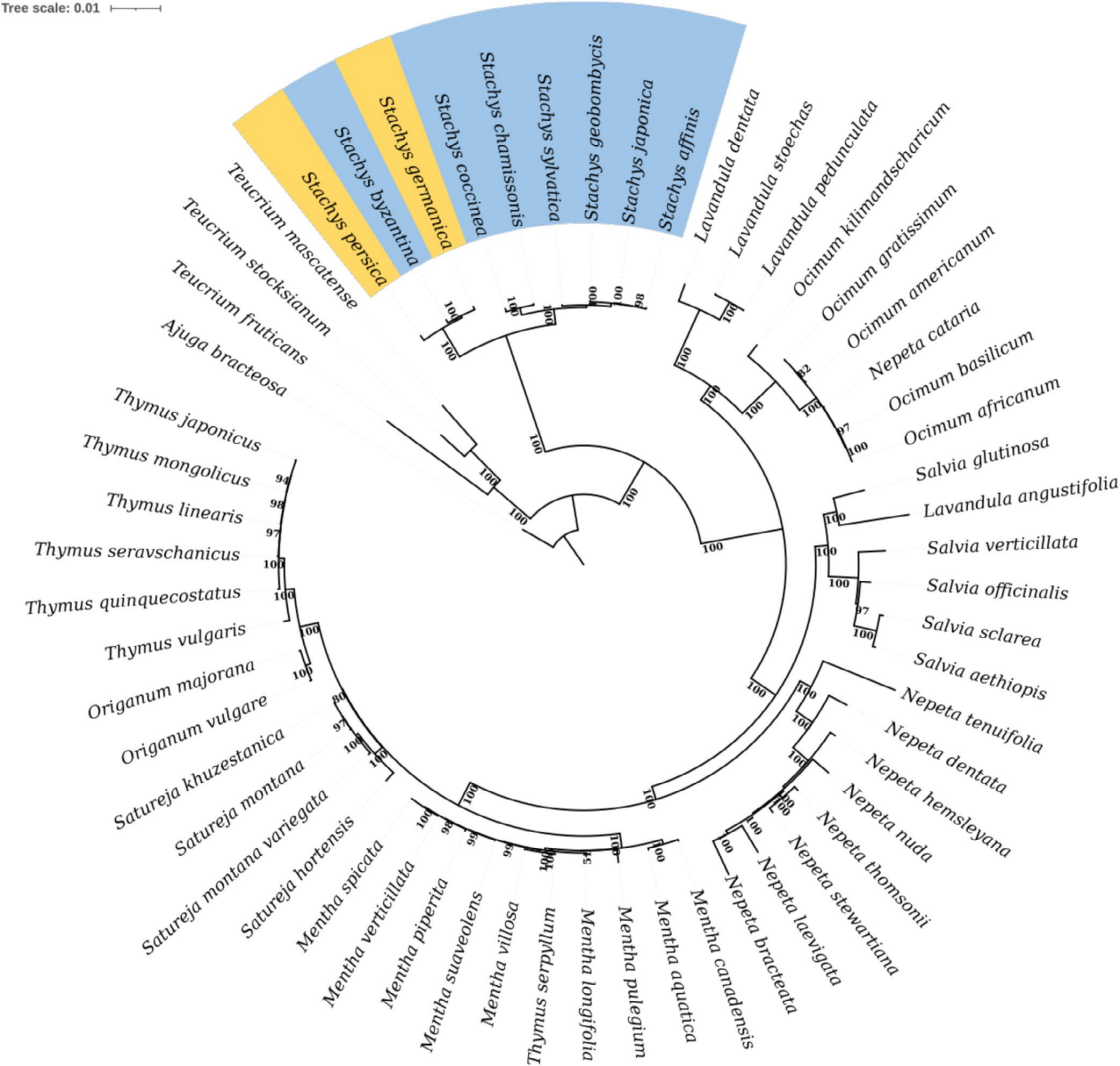
Phylogenetic tree of 58 cp genomes, including 9 *Stachys* species, other species such as *Salvia, Thymus, Menthia, Lavandula, Origanum, Ocimum*, and one outgroup ( *A. bracteosa* ), reconstructed using maximum likelihood in IQ‐TREE under the GTR + Gamma model. The highlighted region emphasizes the position and relationships of the sample sequences from this study, illustrating their clustering with closely related species. Branch support was assessed with 100 bootstrap replicates.

The phylogenetic relationships within the Lamiaceae family, with a specific focus on the genus *Stachys*, were reconstructed using maximum likelihood analysis of complete cp genome sequences. The resulting tree (Figure 6) was robust and well‐resolved, with strong bootstrap support at most nodes. The outgroup, *A. bracteosa* , was placed as expected. The two *Stachys* species sequenced in this study, *S. germanica* and *S. persica* , were firmly embedded within a larger, monophyletic *Stachys* clade with 100% bootstrap support. This clade was resolved as a sister group to the genus *Teucrium*. Within the *Stachys* clade, the species *S. germanica* and *S. byzantina* formed a distinct, strongly supported subclade, which was sister to *S. persica* . Furthermore, the phylogeny delineated a separate, highly supported subclade containing *S. chamissonis* and *S. coccinea* , while species such as *S. sylvatica* , *S. geobombycis*, *S. affinis* , and *S. japonica* formed another distinct lineage. This topology confirms the monophyly of *Stachys* and provides a clear phylogenetic context for the newly sequenced species, indicating a close relationship between *S. germanica* and *S. byzantina* .

**FIGURE 7:**
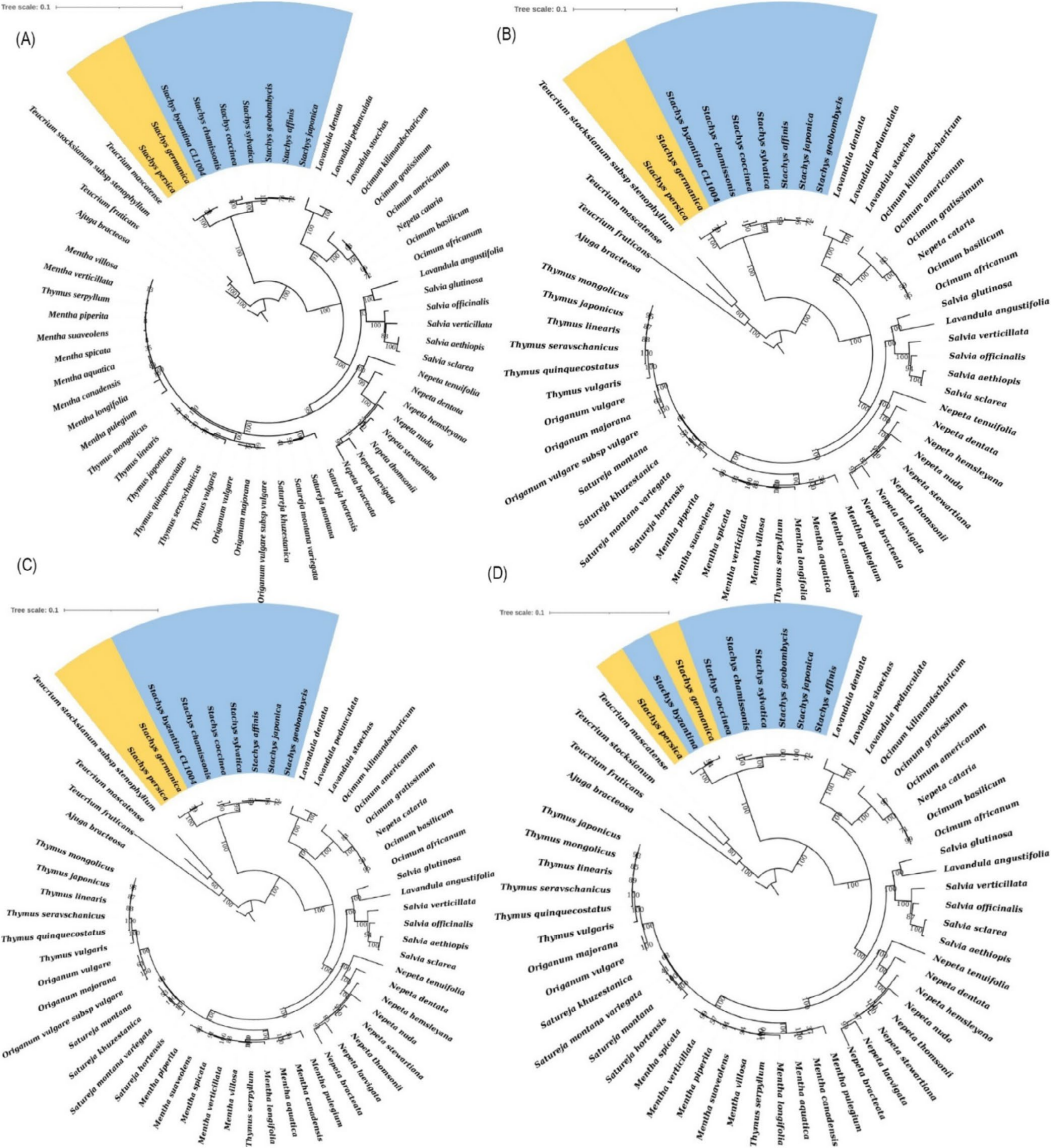
Phylogenetic tree of 58 cp genomes, including 9 *Stachys* species, other species such as *Salvia*, *Thymus*, *Menthia*, *Lavandula*, *Origanum*, *Ocimum*, and one outgroup ( *A. bracteosa* ), reconstructed by *matK* (A), *ycf1* (B), *rpl16* (C) and *matk‐ycf1‐rpl16* (D) using maximum likelihood in IQ‐TREE under the GTR + Gamma model. The highlighted region emphasizes the position and relationships of the sample sequences from this study, illustrating their clustering with closely related species. Branch support was assessed with 100 bootstrap replicates.

To assess the phylogenetic utility of specific genomic regions, we also analyzed the protein‐coding genes *matK*, *rpl16*, and *ycf1*, both individually and in a combined matrix (*matK* + *rpl16* + *ycf1*) (Figure 7). The trees inferred from these barcode regions consistently recovered the monophyly of the genus *Stachys*, corroborating the whole‐plastome result. However, the internal relationships within the *Stachys* clade showed notable variation and a general decrease in bootstrap support compared to the whole‐genome tree. The individual *matK* and *rpl16* gene trees, in particular, presented unstable topologies and poorly resolved relationships among key species, including the placement of *S. persica* and *S. germanica* / *S. byzantina* pair. The combined *matK* + *rpl16* + *ycf1* analysis yielded a more resolved phylogeny that was largely congruent with the whole‐genome topology, but with lower support values for critical internal nodes. These findings demonstrate that while these barcode regions are sufficient for generic‐level identification, the complete cp genome data provide a significantly more powerful and reliable resource for resolving species‐level relationships within the complex genus *Stachys*.

## 4 Discussion

The assembly and characterization of the first complete cp genomes for *S. persica* and *S. germanica* fill a critical genomic void for these important species. They also reinforce established patterns of plastome evolution within the genus and the broader Lamiaceae family. Notably, the availability of these genomes provides essential reference points for future studies on genetic diversity, species identification, and evolutionary adaptations within *Stachys*, a genus with considerable ecological and medicinal significance. Our analysis reveals that the genomes of *S. persica* (150,191 bp) and *S. germanica* (150,238 bp) conform to the canonical angiosperm quadripartite structure and exhibit a high degree of conservation in size, GC content (~38.5%), and gene repertoire (131 genes) relative to other *Stachys* species (Huang et al. 2020; Wang et al. 2020; Wang, Lan, et al. 2024). This GC content is consistent with the general AT‐rich nature of cp genomes across angiosperms, which is thought to reflect mutational biases and structural constraints rather than selective pressures. This profound structural and compositional stability is a well‐documented phenomenon in plant cp genomes. It is attributed to a combination of stringent evolutionary constraints. These include uniparental (typically maternal) inheritance, which reduces opportunities for recombination and homogenizes sequences. A low intrinsic mutation rate compared to the nuclear genome and highly efficient DNA repair mechanisms also contribute to maintaining sequence fidelity (Daniell et al. 2016; Dobrogojski et al. 2020). Additionally, the copy correction effect facilitated by IR regions further stabilizes the genome by promoting sequence homogeneity between the two IR copies, thereby suppressing structural divergence over evolutionary time.

Despite this overarching conservation, evolution manifests in subtle, lineage‐specific variations. The minor difference in total length between *S. persica* and *S. germanica* (47 bp) is typical of the genus, where genome size variation is primarily driven by small expansions or contractions in the LSC region and at the boundaries of IRs. This pattern is not unique to *Stachys* but is a recurrent evolutionary theme across Lamiaceae, as demonstrated in a recent comprehensive study of five key genera (*Lavandula*, *Mentha*, *Ocimum*, *Salvia*, and *Thymus*), where total genome lengths varied from 152,048 to 153,995 bp (Alp Furan et al. 2025). Comparative studies in *Teucrium*, *Salvia*, *Mentha*, and *Satureja* have similarly shown that while the core gene content and order remain static, the lengths of intergenic spacers and the precise positioning of genes at IR/SC junctions are dynamic, serving as primary sources of interspecific variation (Akrami et al. 2025; Diani Gohar and Soorni 2025; Hejazi et al. 2025; Soorni and Golchini 2025). The conserved quadripartite architecture and gene count reported in previous research across diverse Lamiaceae genera strongly corroborates the evolutionary stasis we observe in *Stachys*, where both species possess 131 genes (Alp Furan et al. 2025).

The dynamics of IR/SSC junction shifts represent a microcosm of cp genome evolution, balancing structural plasticity with functional constraint. These shifts, often involving boundary genes such as *ycf1*, *ndhF*, *rps19*, and *trnH‐psbA*, are primarily driven by mechanisms such as slipped‐strand mispairing and intramolecular recombination between IR copies (Hejazi et al. 2025; Niu et al. 2023; Shan et al. 2021; Zhao et al. 2022). A focused analysis of junction sites in five Lamiaceae species visualized these shifts, confirming that IR expansion and contraction lead to variations in the positioning of genes like *rps19* and *rpl2* at the junctions between the quadripartite regions, which serves as a key source of interspecific variation (Alp Furan et al. 2025). However, in rare cases, such as in *Nepeta laevigata*, IR expansion can lead to the pseudogenization of *rps19* and *ycf1*, illustrating how boundary shifts can occasionally impact gene integrity (Niu et al. 2023). This suggests that while most junction variation is tolerated, there is a limit beyond which structural change may impose a functional cost, potentially leading to gene loss or inactivation. The variability in junction positions, even among closely related species, provides a rich source of phylogenetic signal that can resolve shallow evolutionary relationships where sequence divergence alone may be insufficient. For example, specific patterns of *ycf1* truncation or *ndhF* boundary overlap can serve as synapomorphic traits defining species groups or sections within a genus (Wang, Lan, et al. 2024; Wang, Xu, et al. 2024).

The present study characterized the distribution and positional landscape of SSRs within the cp genomes of nine *Stachys* species, with a focus on *S. persica* and *S. germanica* . While differences in repeat motifs and genomic locations were observed between the two focal species, the actual variability of these SSR loci across the full set of nine species was not quantitatively assessed. Such an assessment would require a locus‐by‐locus comparison of repeat length and presence‐absence patterns, which was beyond the scope of this study. Consequently, the utility of the identified cpSSRs as polymorphic markers for population genetics, species authentication, or phylogeographic studies remains to be validated. Future work should systematically evaluate the cross‐species polymorphism of these candidate loci, particularly those located in hypervariable intergenic spacers such as trnE‐UUC‐trnT‐GGU and within the *ycf1* gene.

The analysis of nucleotide diversity across cp genomes of nine *Stachys* species revealed a dual architecture, characterized by deeply conserved core regions alongside highly variable hotspots, a pattern strongly echoed in other Lamiaceae genera such as *Mentha*, *Lavandula*, *Teucrium*, and *Salvia*. Within *Stachys*, our findings paralleled those for *S. geobombycis* (Wang, Lan, et al. 2024), with overall low nucleotide diversity but pronounced heterogeneity. Divergence was concentrated in the single‐copy regions (LSC and SSC), while IR regions showed extreme conservation, a fundamental structural constraint in Lamiaceae (Akrami et al. 2025; Hejazi et al. 2025). IGS regions were the primary reservoirs of diversity. The most variable spacers in our analysis, such as trnN‐GUU–trnR‐ACG, matched those identified by Wang, Lan, et al. (2024) for *S. geobombycis*, indicating lineage‐specific mutation hotspots valuable for species discrimination. Contrasting with Scutellaria and Scutellarioideae, which showed higher overall plastome diversity, especially in the SSC, hyper‐variable loci such as *ndhF*, *ycf1*, *psbA–trnH*, *trnK–rps16* intron, *rbcL–accD*, and *rpl32–trnL* overlapped with hotspots in *Stachys*, indicating broadly conserved regions of accelerated evolution across Lamiaceae. The recurrence of *psbA–trnH* as a hotspot corroborated its role as a universal plant DNA barcode (Hollingsworth et al. 2009; Kress and Erickson 2007). Yet, for closely related taxa in species‐rich genera like *Stachys*, lineage‐specific spacers offered higher discriminatory power than a single universal marker. Applied DNA barcoding studies in medicinal Lamiaceae supported this. Thakur et al. (2021) showed matK outperformed *psbA–trnH* and *trnL* for distinguishing *Mentha*, *Ocimum*, and *Plectranthus* species. This aligns with the observation in the present study that *matK* was among the most polymorphic protein‐coding genes in *Stachys* (Thakur et al. 2021). Conversely, studies on *Lavandula* (Hind et al. 2018) showed plastid loci like *rbcL* and *psbA–trnH* could be insufficient due to hybridization, underscoring that lineage‐specific hotspots identified through comparative genomics were essential for robust resolution. In *Stachys*, essential functional elements, including many tRNA, photosynthetic, and ribosomal protein genes, were entirely invariant, mirroring *S. geobombycis* (Wang, Lan, et al. 2024) and reflecting strong purifying selection. Among protein‐coding genes, *matK* and *ycf1* were the most polymorphic, a pattern repeated in *Teucrium*, *Salvia*, *Mentha*, and *Scutellaria* (Akrami et al. 2025; Hejazi et al. 2025; Salmaki et al. 2016; Soorni and Golchini 2025).

Despite the power of whole‐plastome data to resolve phylogenetic relationships and identify hypervariable markers, an important caveat must be considered when interpreting species‐level trees derived solely from cp genomes. The cp genome is maternally inherited and can be transferred between species through hybridization, a phenomenon known as chloroplast capture (Filip and Skuza 2021; Stegemann et al. 2012). This can lead to discordance between cp and species trees, particularly among recently diverged or hybridizing lineages. In *Stachys*, historical hybridization can be accrued, but no experimental or published evidence has directly documented cp capture in the genus. Consequently, we cannot rule out the possibility that some species‐level relationships inferred from our plastome data may be influenced by cp sharing.

Beyond nucleotide diversity, our detection of positive selection in the photosynthetic gene *petB* addresses a separate but equally important evolutionary question. While the conserved and invariant regions discussed above reflect strong purifying selection across most of the plastome, the *petB* gene stands out as an exception. This gene encodes cytochrome *b*_*6*_, a core subunit of the cytochrome *b*_*6*_ *f* complex that mediates electron transfer between photosystems II and I. The finding of positive selection in a chloroplast gene is notable because such genes are typically under strong purifying selection. Similar signatures of adaptive evolution in *petB* have been reported in *Mangifera* (Tang et al. 2025), *Gossypium* (Wu et al. 2018), *Brassica oleracea var. alboglabra* (Wang et al. 2023), and *Balanites aegyptiaca* (AL‐Juhani et al. 2022). The repeated emergence of adaptive signals in this gene across phylogenetically distant taxa suggests that fine‐tuning electron transport chain kinetics may represent an evolutionarily recurrent strategy for coping with ecological stressors such as variable light intensity, temperature extremes, or oxidative pressure. In *Stachys*, the two positively selected codons reside at the N‐terminus of the mature protein (sites 1 and 2), a region potentially involved in protein folding, membrane insertion, or interaction with the *petD* subunit. While the functional consequences of these specific amino acid changes remain to be tested experimentally, their location at a structurally sensitive interface suggests that even conserved photosynthetic complexes can undergo lineage‐specific adaptive refinement.

## 5 Conclusion

This study provides the first comprehensive cp genomic resources for two medicinally significant *Stachys* species, *S. persica* and *S. germanica* . Through whole‐plastome sequencing, we have significantly advanced the molecular systematics of this complex genus. Our comparative analysis revealed a strongly conserved genomic structure across the genus, with lineage‐specific variation primarily driven by expansions/contractions at IR‐SC boundaries and mutations in intergenic spacers. The cpSSRs identified in this study, particularly those showing length variation between *S. persica* and *S. germanica* , represent candidate markers, though their broader utility across the genus requires empirical validation in future studies. The identification of hypervariable regions, particularly the trnL‐trnF spacer and the matK and *ycf1* genes, provides robust, high‐resolution candidate DNA barcodes that outperform standard markers for species discrimination within *Stachys*. Furthermore, the detection of positive selection acting on the *petB* gene suggests adaptive evolution in a core component of the photosynthetic electron transport chain. Most critically, the phylogenomic reconstruction using complete cp genome sequences established a robust evolutionary framework, definitively resolving the close relationship between *S. germanica* and *S. byzantina* and clarifying the position of *S. persica* . However, broader taxonomic sampling will be required to fully resolve infrageneric relationships across the entire genus. These findings not only fill a critical data gap but also establish a powerful genomic foundation for future studies in taxonomy, conservation, phytochemistry, and the authentication of medicinal material within this important genus.

## Author Contributions

**Fatemeh Sadat Ghotbi:** formal analysis (equal), investigation (equal), methodology (equal), writing – review and editing (equal). **Aboozar Soorni:** conceptualization (equal), formal analysis (equal), funding acquisition (equal), project administration (equal), validation (equal), visualization (equal), writing – original draft (equal), writing – review and editing (equal).

## Funding

The authors have nothing to report.

## Ethics Statement

The authors declared that experimental research works on the plants described in this paper comply with institutional, national, and international guidelines. This article does not involve any endangered or protected species.

## Consent

The authors have nothing to report.

## Conflicts of Interest

The authors declare no conflicts of interest.

## Data Availability

The assembled and annotated genome is accessible in NCBI database under the research accessions PX260186 and PX260185.

## References

[ece373618-bib-0001] Akrami, A. M. , S. Meratian Esfahani , and A. Soorni . 2025. “Decoding the Chloroplast Genomes of Five Iranian Salvia Species: Insights Into Genomic Structure, Phylogenetic Relationships, and Molecular Marker Development.” BMC Genomics 26: 545. 40448040 10.1186/s12864-025-11729-0 PMC12123993

[ece373618-bib-0002] AL‐Juhani, W. S. , S. A. Alharbi , N. M. Al Aboud , and A. Y. Aljohani . 2022. “Complete Chloroplast Genome of the Desert Date ( *Balanites aegyptiaca* L.) Del Comparative Analysis, and Phylogenetic Relationships Among the Members of Zygophyllaceae.” BMC Genomics 23: 626. 10.1186/s12864-022-08850-9. 36045328 PMC9434970

[ece373618-bib-0003] Alp Furan, M. , F. Yildiz , and O. Kaya . 2025. “Exploring the Complete Chloroplast Genomes of Key Lamiaceae Species Uncovers the Secrets of Evolutionary Dynamics and Phylogenetic Relationships.” Journal of Plant Growth Regulation 44: 1–14.

[ece373618-bib-0004] Amiryousefi, A. , J. Hyvönen , and P. Poczai . 2018. “IRscope: An Online Program to Visualize the Junction Sites of Chloroplast Genomes.” Bioinformatics 34: 3030–3031. 29659705 10.1093/bioinformatics/bty220

[ece373618-bib-0005] Berumen Cornejo, A. M. , C. Lindqvist , E. M. Perez Molphe Balch , and M. E. Siqueiros Delgado . 2017. “Phylogeny of the *Stachys coccinea* (Lamiaceae) Complex Based on Molecular and Morphological Data.” Systematic Botany 42: 484–493.

[ece373618-bib-0006] Bolger, A. M. , M. Lohse , and B. Usadel . 2014. “Trimmomatic: A Flexible Trimmer for Illumina Sequence Data.” Bioinformatics 30: 2114–2120. 10.1093/bioinformatics/btu170. 24695404 PMC4103590

[ece373618-bib-0007] Capella‐Gutiérrez, S. , J. M. Silla‐Martínez , and T. Gabaldón . 2009. “trimAl: A Tool for Automated Alignment Trimming in Large‐Scale Phylogenetic Analyses.” Bioinformatics 25: 1972–1973. 10.1093/bioinformatics/btp348. 19505945 PMC2712344

[ece373618-bib-0008] Daniell, H. , C.‐S. Lin , M. Yu , and W.‐J. Chang . 2016. “Chloroplast Genomes: Diversity, Evolution, and Applications in Genetic Engineering.” Genome Biology 17: 1–29. 27339192 10.1186/s13059-016-1004-2 PMC4918201

[ece373618-bib-0009] Diani Gohar, S. , and A. Soorni . 2025. “Plastome Diversity and Phylogenomic Analysis of Satureja (Lamiaceae): Uncovering Evolutionary Patterns and Diagnostic Markers.” Planta 262: 149. 41204963 10.1007/s00425-025-04875-y

[ece373618-bib-0010] Dobrogojski, J. , M. Adamiec , and R. Luciński . 2020. “The Chloroplast Genome: A Review.” Acta Physiologiae Plantarum 42: 98.

[ece373618-bib-0011] Dong, W. , J. Liu , J. Yu , L. Wang , and S. Zhou . 2012. “Highly Variable Chloroplast Markers for Evaluating Plant Phylogeny at Low Taxonomic Levels and for DNA Barcoding.” PLoS One 7: e35071. 22511980 10.1371/journal.pone.0035071 PMC3325284

[ece373618-bib-0012] Dong, W. , C. Xu , C. Li , et al. 2015. “ycf1, the Most Promising Plastid DNA Barcode of Land Plants.” Scientific Reports 5: 8348. 25672218 10.1038/srep08348 PMC4325322

[ece373618-bib-0013] Dündar, E. , E. Akçiçek , T. Dirmenci , and Ş. Akgün . 2013. “Phylogenetic Analysis of the Genus Stachys Sect. Eriostomum (Lamiaceae) in Turkey Based on Nuclear Ribosomal ITS Sequences.” Turkish Journal of Botany 37: 14–23.

[ece373618-bib-0014] Edgar, R. C. 2004. “MUSCLE: Multiple Sequence Alignment With High Accuracy and High Throughput.” Nucleic Acids Research 32: 1792–1797. 10.1093/nar/gkh340. 15034147 PMC390337

[ece373618-bib-0015] Filip, E. , and L. Skuza . 2021. “Horizontal Gene Transfer Involving Chloroplasts.” International Journal of Molecular Sciences 22: 4484. 10.3390/ijms22094484. 33923118 PMC8123421

[ece373618-bib-0016] Gao, F. , C. Chen , D. A. Arab , Z. Du , Y. He , and S. Y. W. Ho . 2019. “EasyCodeML: A Visual Tool for Analysis of Selection Using CodeML.” Ecology and Evolution 9: 3891–3898. 31015974 10.1002/ece3.5015 PMC6467853

[ece373618-bib-0017] Goren, A. C. , F. Piozzi , E. Akcicek , et al. 2011. “Essential Oil Composition of Twenty‐Two Stachys Species (Mountain Tea) and Their Biological Activities.” Phytochemistry Letters 4: 448–453.

[ece373618-bib-0018] Greiner, S. , P. Lehwark , and R. Bock . 2019. “OrganellarGenomeDRAW (OGDRAW) Version 1.3. 1: Expanded Toolkit for the Graphical Visualization of Organellar Genomes.” Nucleic Acids Research 47: W59–W64. 30949694 10.1093/nar/gkz238 PMC6602502

[ece373618-bib-0019] Grujic‐Jovanovic, S. , H. D. Skaltsa , P. Marin , and M. Sokovic . 2004. “Composition and Antibacterial Activity of the Essential Oil of Six Stachys Species From Serbia.” Flavour and Fragrance Journal 19: 139–144.

[ece373618-bib-0020] Güner, A. , N. Özhatay , T. Ekim , K. H. C. Başer , I. C. Hedge , and I. C. Hedge . 2000. Flora of Turkey and the East Aegean Islands. Vol. 11. Edinburgh University Press.

[ece373618-bib-0021] Hejazi, F. A. , P. Mohammadi , and A. Soorni . 2025. “Comparative Chloroplast Genomics of Teucrium Species Reveals Genome Evolution, Phylogenetic Relationships, and Candidate Molecular Markers.” Scientific Reports 15: 44318. 41429813 10.1038/s41598-025-29339-x PMC12722754

[ece373618-bib-0022] Hind, K. R. , A. M. Adal , T. M. Upson , and S. S. Mahmoud . 2018. “An Assessment of Plant DNA Barcodes for the Identification of Cultivated Lavandula (Lamiaceae) Taxa.” Biocatalysis and Agricultural Biotechnology 16: 459–466.

[ece373618-bib-0023] Hollingsworth, M. L. , A. Andra Clark , L. L. Forrest , et al. 2009. “Selecting Barcoding Loci for Plants: Evaluation of Seven Candidate Loci With Species‐Level Sampling in Three Divergent Groups of Land Plants.” Molecular Ecology Resources 9: 439–457. 21564673 10.1111/j.1755-0998.2008.02439.x

[ece373618-bib-0024] Huang, L. , H. Yu , Z. Wang , and W. Xu . 2024. “CPStools: A Package for Analyzing Chloroplast Genome Sequences.” iMetaOmics 1: e25. 41676121 10.1002/imo2.25 PMC12806284

[ece373618-bib-0025] Huang, W. , X. Gao , Y. Zhang , C. Jin , and X. Wang . 2020. “The Complete Chloroplast Genome Sequence of *Stachys sieboldii* Miquel.(Labiatae), a Kind of Vegetable Crop and Chinese Medicinal Material Plant.” Mitochondrial DNA Part B Resources 5: 1832–1833.

[ece373618-bib-0026] Jaswal, S. , A. K. Srivastava , A. Ballal , and S. K. Sandur . 2025. “Chloroplast Engineering for Enhancing Photosynthetic Efficiency and Agronomic Traits.” Trends in Biotechnology. 10.1016/j.tibtech.2025.09.011 41068042

[ece373618-bib-0027] Jin, J.‐J. , W.‐B. Yu , J.‐B. Yang , et al. 2020. “GetOrganelle: A Fast and Versatile Toolkit for Accurate De Novo Assembly of Organelle Genomes.” Genome Biology 21: 1–31. 10.1186/s13059-020-02154-5 PMC7488116 32912315

[ece373618-bib-0028] Kadereit, J. W. 2004. Flowering Plants· Dicotyledons: Lamiales (Except Acanthaceae Including Avicenniaceae). Springer Science & Business Media.

[ece373618-bib-0029] Khanavi, M. , A. Hadjiakhoondi , G. Amin , Y. Amanzadeh , A. Rustaiyan , and A. Shafiee . 2004. “Comparison of the Volatile Composition of Stachys Persica Gmel. and *Stachys byzantina* C. Koch. Oils Obtained by Hydrodistillation and Steam Distillation.” Zeitschrift Für Naturforschung. Section C 59: 463–467. 10.1515/znc-2004-7-802 15813362

[ece373618-bib-0030] Kharazian, N. , S. Rahimi , and B. Shiran . 2015. “Genetic Diversity and Morphological Variability of Fifteen Stachys (Lamiaceae) Species From Iran Using Morphological and ISSR Molecular Markers.” Biologia (Bratisl) 70: 438–452.

[ece373618-bib-0031] Kochieva, E. Z. , N. N. Ryzhova , M. P. Legkobit , and N. V. Khadeeva . 2006. “RAPD and ISSR Analyses of Species and Populations of the Genus Stachys.” Russian Journal of Genetics 42: 723–727. 16915917

[ece373618-bib-0032] Kress, W. J. , and D. L. Erickson . 2007. “A Two‐Locus Global DNA Barcode for Land Plants: The Coding rbcL Gene Complements the Non‐Coding trnH‐psbA Spacer Region.” PLoS One 2: e508. 17551588 10.1371/journal.pone.0000508 PMC1876818

[ece373618-bib-0033] Langmead, B. , C. Trapnell , M. Pop , and S. L. Salzberg . 2009. “Ultrafast and Memory‐Efficient Alignment of Short DNA Sequences to the Human Genome.” Genome Biology 10: R25. 10.1186/gb-2009-10-3-r25. 19261174 PMC2690996

[ece373618-bib-0034] Lashgargahi, Z. , and A. Shafaghat . 2017. “Volatile Constituents of Essential Oils Isolated From Fresh and Dried Stachys Lavandulifolia Vahl. And *Stachys byzantina* C. Koch. Two Lamiaceae From North‐West Iran.” Journal of Essential Oil‐Bearing Plants 20: 1302–1309.

[ece373618-bib-0035] Letunic, I. , and P. Bork . 2021. “Interactive Tree of Life (iTOL) v5: An Online Tool for Phylogenetic Tree Display and Annotation.” Nucleic Acids Research 49: W293–W296. 10.1093/nar/gkab301. 33885785 PMC8265157

[ece373618-bib-0036] Li, H. , B. Handsaker , A. Wysoker , et al. 2009. “The Sequence Alignment/Map Format and SAMtools.” Bioinformatics 25: 2078–2079. 10.1093/bioinformatics/btp352. 19505943 PMC2723002

[ece373618-bib-0037] Lindqvist, C. , and V. A. Albert . 2002. “Origin of the Hawaiian Endemic Mints Within North American Stachys (Lamiaceae).” American Journal of Botany 89: 1709–1724. 21665597 10.3732/ajb.89.10.1709

[ece373618-bib-0038] Mulligan, G. A. , and D. B. Munro . 1989. “Taxonomy of Species of North American Stachys (Labiatae) Found North of Mexico.”

[ece373618-bib-0039] Nguyen, L.‐T. , H. A. Schmidt , A. von Haeseler , and B. Q. Minh . 2015. “IQ‐TREE: A Fast and Effective Stochastic Algorithm for Estimating Maximum‐Likelihood Phylogenies.” Molecular Biology and Evolution 32: 268–274. 10.1093/molbev/msu300. 25371430 PMC4271533

[ece373618-bib-0040] Niu, Y. , Q. Qin , Y. Dong , X. Wang , S. Zhang , and Z. Mu . 2023. “Chloroplast Genome Structure and Phylogenetic Analysis of 13 Lamiaceae Plants in Tibet.” Frontiers in Bioscience 28: 110. 10.31083/j.fbl2806110 37395020

[ece373618-bib-0041] Ravi, V. , J. P. Khurana , A. K. Tyagi , and P. Khurana . 2008. “An Update on Chloroplast Genomes.” Plant Systematics and Evolution 271: 101–122.

[ece373618-bib-0042] Razazi, N. , A. A. Jafari , Z. Khodarahmpour , and S. Sadat . 2023. “Variation of Yield, Morphological Traits, and Essential Oil in Populations of Five Species of Stachys L. in Iran.” Journal of Agricultural Science and Technology 25: 1179–1191.

[ece373618-bib-0043] Rechinger, K. H. 1982. Flora Iranica: Flora Des Iranischen Hochlandes Und Der Umrahmenden Gebirge. Vol. 150, 292–313. Akademische Druck‐ u. Verlagsanstalt.

[ece373618-bib-0044] Roy, T. , T.‐H. Chang , T. Lan , and C. Lindqvist . 2013. “Phylogeny and Biogeography of New World Stachydeae (Lamiaceae) With Emphasis on the Origin and Diversification of Hawaiian and South American Taxa.” Molecular Phylogenetics and Evolution 69: 218–238. 23769956 10.1016/j.ympev.2013.05.023

[ece373618-bib-0045] Rozas, J. , A. Ferrer‐Mata , J. C. Sánchez‐DelBarrio , et al. 2017. “DnaSP 6: DNA Sequence Polymorphism Analysis of Large Data Sets.” Molecular Biology and Evolution 34: 3299–3302. 29029172 10.1093/molbev/msx248

[ece373618-bib-0046] Salmaki, Y. , G. Heubl , and M. Weigend . 2019. “Towards a New Classification of Tribe Stachydeae (Lamiaceae): Naming Clades Using Molecular Evidence.” Botanical Journal of the Linnean Society 190: 345–358.

[ece373618-bib-0047] Salmaki, Y. , Z. Jamzad , S. Zarre , and C. Bräuchler . 2008. “Pollen Morphology of Stachys (Lamiaceae) in Iran and Its Systematic Implication.” Flora ‐ Morphology, Distribution, Functional Ecology of Plants 203: 627–639.

[ece373618-bib-0048] Salmaki, Y. , S. Kattari , G. Heubl , and C. Bräuchler . 2016. “Phylogeny of Non‐Monophyletic Teucrium (Lamiaceae: Ajugoideae): Implications for Character Evolution and Taxonomy.” Taxon 65: 805–822.

[ece373618-bib-0049] Salmaki, Y. , S. Zarre , R. Govaerts , and C. Bräuchler . 2012. “A Taxonomic Revision of the Genus Stachys (Lamiaceae: Lamioideae) in Iran.” Botanical Journal of the Linnean Society 170: 573–617.

[ece373618-bib-0050] Seyedipour, S. , Y. Salmaki , and C. Xiang . 2017. “Molecular Phylogeny of Scutellaria (Lamiaceae; Scutellarioideae) in Iranian Highlands Inferred From nrITS and trnL‐F Sequences.” Progress in Biological Sciences 7: 169–181.

[ece373618-bib-0051] Shan, Y. , X. Pei , S. Yong , et al. 2021. “Analysis of the Complete Chloroplast Genomes of Scutellaria Tsinyunensis and Scutellaria Tuberifera (Lamiaceae).” Mitochondrial DNA Part B Resources 6: 2672–2680. 34435116 10.1080/23802359.2021.1920491 PMC8381982

[ece373618-bib-0052] Shi, L. , H. Chen , M. Jiang , et al. 2019. “CPGAVAS2, an Integrated Plastome Sequence Annotator and Analyzer.” Nucleic Acids Research 47: W65–W73. 31066451 10.1093/nar/gkz345 PMC6602467

[ece373618-bib-0053] Soorni, A. , and M. M. Golchini . 2025. “Complete Chloroplast Genome of *Mentha aquatica* Reveals Hypervariable Regions and Resolves Phylogenetic Position Within the Genus Mentha.” Molecular Biology Reports 52: 677. 40622487 10.1007/s11033-025-10789-5

[ece373618-bib-0054] Stegemann, S. , M. Keuthe , S. Greiner , and R. Bock . 2012. “Horizontal Transfer of Chloroplast Genomes Between Plant Species.” Proceedings of the National Academy of Sciences 109: 2434–2438. 10.1073/pnas.1114076109. PMC3289295 22308367

[ece373618-bib-0055] Tamura, K. , G. Stecher , D. Peterson , A. Filipski , and S. Kumar . 2013. “MEGA6: Molecular Evolutionary Genetics Analysis Version 6.0.” Molecular Biology and Evolution 30: 2725–2729. 24132122 10.1093/molbev/mst197 PMC3840312

[ece373618-bib-0056] Tang, Y. , X. Yang , S. Luo , et al. 2025. “Comparative and Phylogenetic Analysis of Complete Chloroplast Genomes of Five Mangifera Species.” Genes (Basel) 16: 666. 10.3390/genes16060666. 40565558 PMC12192436

[ece373618-bib-0057] Thakur, V. V. , N. Tripathi , and S. Tiwari . 2021. “DNA Barcoding of Some Medicinally Important Plant Species of Lamiaceae Family in India.” Molecular Biology Reports 48: 3097–3106. 33913093 10.1007/s11033-021-06356-3

[ece373618-bib-0058] Tillich, M. , P. Lehwark , T. Pellizzer , et al. 2017. “GeSeq–Versatile and Accurate Annotation of Organelle Genomes.” Nucleic Acids Research 45: W6–W11. 28486635 10.1093/nar/gkx391 PMC5570176

[ece373618-bib-0059] Tomou, E.‐M. , C. Barda , and H. Skaltsa . 2020. “Genus Stachys: A Review of Traditional Uses, Phytochemistry and Bioactivity.” Medicine 7: 63. 10.3390/medicines7100063 PMC7601302 33003416

[ece373618-bib-0060] Tomou, E.‐M. , A. Karioti , G. Tsirogiannidis , N. Krigas , and H. Skaltsa . 2023. “Metabolic Characterization of Four Members of the Genus *Stachys* L. (Lamiaceae).” Agronomy 13: 2624.

[ece373618-bib-0061] Tundis, R. , L. Peruzzi , and F. Menichini . 2014. “Phytochemical and Biological Studies of Stachys Species in Relation to Chemotaxonomy: A Review.” Phytochemistry 102: 7–39. 24661611 10.1016/j.phytochem.2014.01.023

[ece373618-bib-0062] Vaidya, G. , D. J. Lohman , and R. Meier . 2011. “SequenceMatrix: Concatenation Software for the Fast Assembly of Multi‐Gene Datasets With Character Set and Codon Information.” Cladistics 27: 171–180. 10.1111/j.1096-0031.2010.00329.x. 34875773

[ece373618-bib-0063] Wang, M. , Q. Zhao , D. Jiang , and Z. Wang . 2020. “Complete Chloroplast Genome Sequence of Stachys Japonica (Labiatae).” Mitochondrial DNA Part B Resources 5: 2675–2676. 33457900 10.1080/23802359.2020.1787263 PMC7782854

[ece373618-bib-0064] Wang, R. , Z. Lan , Y. Luo , and Z. Deng . 2024. “The Complete Chloroplast Genome of Stachys Geobombycis and Comparative Analysis With Related Stachys Species.” Scientific Reports 14: 8523. 38609472 10.1038/s41598-024-59132-1 PMC11014926

[ece373618-bib-0065] Wang, Y. , Q. Liang , C. Zhang , et al. 2023. “Sequencing and Analysis of Complete Chloroplast Genomes Provide Insight Into the Evolution and Phylogeny of Chinese Kale ( *Brassica oleracea var. alboglabra* ).” International Journal of Molecular Sciences 24: 10287. 10.3390/ijms241210287. 37373434 PMC10299174

[ece373618-bib-0066] Wang, Y. , C. Xu , X. Guo , et al. 2024. “Phylogenomics Analysis of Scutellaria (Lamiaceae) of the World.” BMC Biology 22: 185. 39218872 10.1186/s12915-024-01982-2 PMC11367873

[ece373618-bib-0067] Wick, R. R. , M. B. Schultz , J. Zobel , and K. E. Holt . 2015. “Bandage: Interactive Visualization of De Novo Genome Assemblies.” Bioinformatics 31: 3350–3352. 26099265 10.1093/bioinformatics/btv383 PMC4595904

[ece373618-bib-0068] Wu, Y. , F. Liu , D.‐G. Yang , et al. 2018. “Comparative Chloroplast Genomics of Gossypium Species: Insights Into Repeat Sequence Variations and Phylogeny.” Frontiers in Plant Science 9: 9–2018. 29619041 10.3389/fpls.2018.00376 PMC5871733

[ece373618-bib-0069] Zhang, Y. , L. Tian , and C. Lu . 2023. “Chloroplast Gene Expression: Recent Advances and Perspectives.” Plant Communications 4: 100611. 37147800 10.1016/j.xplc.2023.100611 PMC10504595

[ece373618-bib-0070] Zhao, F. , Y.‐P. Chen , Y. Salmaki , et al. 2021. “An Updated Tribal Classification of Lamiaceae Based on Plastome Phylogenomics.” BMC Biology 19: 2. 33419433 10.1186/s12915-020-00931-z PMC7796571

[ece373618-bib-0071] Zhao, W. , L. Guo , Y. Yang , et al. 2022. “Complete Chloroplast Genome Sequences of Phlomis Fruticosa and Phlomoides Strigosa and Comparative Analysis of the Genus Phlomis Sensu Lato (Lamiaceae).” Frontiers in Plant Science 13: 1022273. 36388530 10.3389/fpls.2022.1022273 PMC9650320

